# Complications post renal transplantation: literature focus on BK virus nephropathy and diagnostic tools actually available

**DOI:** 10.1186/1743-422X-5-38

**Published:** 2008-03-03

**Authors:** Monica Mischitelli, Anna Bellizzi, Elena Anzivino, Daniela Fioriti, Renzo Boldorini, Umberto Miglio, Fernanda Chiarini, Franco Di Monaco, Valeria Pietropaolo

**Affiliations:** 1 Department of Public Health Sciences, "La Sapienza" University, Rome, Italy; 2 Department of Medical Sciences, Faculty of Medicine, University Amedeo Avogadro of East Piedmont, Novara, Italy; 3 Department of Urology, "La Sapienza" University, Rome, Italy

## Abstract

Clinical diagnosis of kidney transplants related illnesses is not a simple task. Several studies were conducted to define diseases and complications after renal transplantation, but there are no comprehensive guidelines about diagnostic tools for their prevention and detection.

The Authors of this review looked for the medical literature and pertinent publications in particular to understand the role of Human Polyomavirus BK (BKV) in renal failure and to recognize analytical techniques for BK virus associated nephropathy (BKVAN) detection.

## Introduction

Clinical diagnosis of kidney transplants related illnesses is not a simple task. Several studies were conducted to define diseases and complications after renal transplantation, but there are no comprehensive guidelines about diagnostic tools for their prevention and detection.

The Authors of this review looked for the medical literature and pertinent publications in particular to understand the role of Human Polyomavirus BK (BKV) in renal failure and to recognize analytical techniques for BK virus associated nephropathy (BKVAN) detection. For reviewing we used Medline and recent pertinent bibliographies.

Kidney pathologies in renal transplants are associated with graft function, immunosuppressive drugs and infections [1]. Moreover cardiovascular, bone and bone marrow diseases, metabolism dysfunctions and cancers could affect these patients [2,3]. Graft function is the most important parameter in evaluation of the allograft status; acute rejection, obstruction, renal artery stenosis could influence renal function resulting in graft dysfunctions and ultimately in chronic renal allograft failure [1,4,5]. Persistent urinary protein excretion and hyperlipidemia are associated with acute rejection, in particular heavy proteinuria has important consequences for extracellular fluid volume regulation and demonstrate the rapid deterioration of renal function associated with pathologic glomerular lesions [6,7]. Serum creatinine levels and urine protein/creatinine ratio (total protein excretion) should be used to screen for changes in renal function. Acute allograft rejection could be also due to interstitial infiltrates and mild tubulitis that unfortunately are clinically silent and could be detected only by immunohistochemistry (IHC) [1].

## Immunosuppression therapy

The morbidity and mortality rates associated with renal transplantation and the use of immunosuppressive medications are high. Conventional immunosuppression is based on azathioprine, nevertheless, other immunosuppressive drugs, such as cyclosporine A (CsA), tacrolimus, sirolimus, mycophenolate-mofetil (MMF) and corticosteroids are used [1,8]. To reduce adverse effects of immunosuppressive therapies, it is strongly recommended to monitor routinely blood level of CsA, tacrolimus and sirolimus. The nephrotoxicity associated with azathioprine and MMF is monitored by assessing hemoglobin levels, hematocrit value and white blood cell counts at least weekly for months 1 to 2, every 2 week for months 3 to 4, monthly for months 4 to 12, and then every 3 to 6 months [1,8-12]. Finally toxicity related to corticosteroids is monitored periodically by controlling blood pressure, lipoprotein levels and blood glucose levels [8,11]. Compared with conventional immunosuppression with azathioprine, CsA reduced the incidence of acute rejection and prolonged graft survival but caused chronic tubulointerstitial atrophy and fibrosis that are difficult to distinguish from chronic allograft nephropathy attributable to other causes [1,13]. Instead the role of acute and chronic tacrolimus nephrotoxicity in graft failure is unclear. However the incidence of renal toxicity is roughly proportional to tacrolimus doses and its blood levels [14]. In the other hand sirolimus seems to be efficacious in preventing acute rejection when used in place of, or in combination with, CsA. However very few studies have been conducted to determine the relationship between blood levels of sirolimus and either acute rejection or toxicity [10]. Regarding azathioprine and MMF, hematologic and gastrointestinal toxicities are usually dose-related and respond to dose reductions [12]. Moreover MMF causes leukopenia in renal transplants. Finally clinical signs of corticosteroid toxicity, which are observed relatively soon after the initiation of prednisone treatment, include skin changes, hypertension, peptic ulcer disease and myopathy [8].

## Human Poliomavirus BK and BKVAN

Viral infections cause several complications in renal transplants that are closely related with the immunosuppressive therapy. On the basis of literature data, viruses implicated in graft failure we could number Varicella zoster, Cytomegalovirus, Influenza A and B, Hepatitis B and C and human Poliomavirus BK and JC [15-18]. In particular BK virus, described for the first time in a transplant recipient, has a remarkable tropism for the genitourinary tract, in fact BKVAN are recognized as an important cause of late allograft failure [19].

BKV is ubiquitous in human populations worldwide. BKV infects young children and the seroprevalence is 70%–80% in adults [20,21]. Serologic surveys of populations, using hemagglutination inhibition assay for the detection of antibodies, indicate that seroconversion takes place early in life, at 5–7 years of age [20,21]. Primary infection is usually inapparent and only occasionally may be accompanied by mild respiratory illness or urinary tract disease. During primary infection viremia occurs and the virus spreads to several organs of the infected individual where it remains in a latent state. After the initial infection, the virus disseminates and establishes a persistent infection in the urinary tract and maybe in lymphocytes [20,22,23].

**Figure 1:**
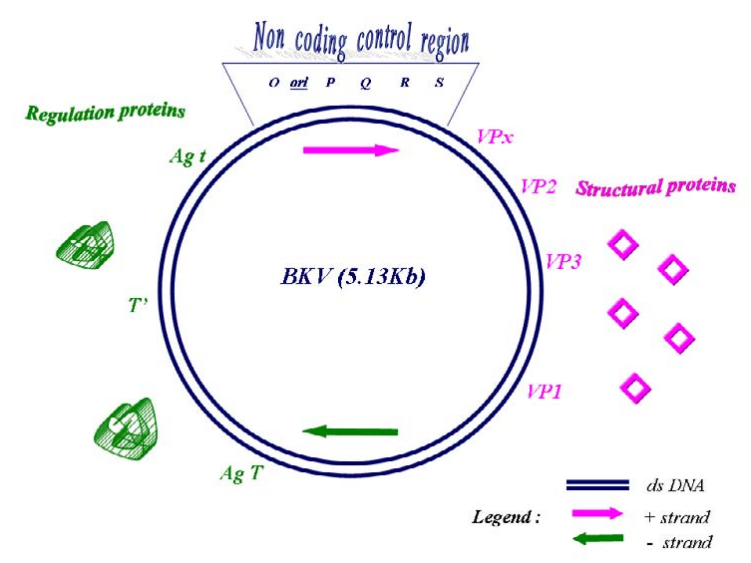
Schematic representation of the gene organization in the BK virus (BKV) genome. The double circle represents the double stranded DNA genomes. The genome is divided into three regions. The early region encodes three regulatory proteins (Agt, AgT, T'). The late region specifies four structural proteins and agnoprotein (VP1, VP2, VP3, VPx). The non-coding control region contains the elements for the control of viral DNA replication (ori) and viral gene expression. The arrows indicate the positive and negative strands according to the direction of viral transcription (24).

The complete genome of BKV contains 5,153 bp and it is functionally divided into three regions: the early, the late, and the transcriptional control region (TCR). The first region codes for the small and large T-antigens (t-Ag and T-Ag), the second region codes for the viral capsid proteins VP1-VP2-VP3 and agno-protein, and the last region (TCR) contains the transcriptional control elements for both "early" and "late" gene expression [24] Primary transcripts are required for viral replication, in particular T-Ag promotes unwinding of the double helix and recruitment of cellular proteins required for DNA synthesis whereas in non permissive cells it is involved in neoplastic transformation [24,25] (Fig. 1). Late transcripts encode for viral capside proteins and agnoprotein, that has a critical role in the regulation of viral gene expression and replication, and in the modulation of certain important host cell functions including cell cycle progression and DNA repair [26]. TCR contains the origin of replication and it is arbitrarily divided into four box alphabetically designated P, Q, R and S. These sequence blocks serve as regulatory regions, or enhancer elements believed to contain several transcription factor binding sites involved in the modulation of viral transcription [24,27,28]. It is not known that genetic alterations are essential for the pathogenesis associated with BKV after kidney transplantation, nevertheless BK-strains with rearranged TCR have been particularly described in subjects under immunosuppressive therapies [24,29,30]. In renal transplants BKV infection may be transmitted via the donor organ, may be acquired in the community or latent BKV could reactivate [31,32]. The incidence of allograft failure has ranged from 15 to 50% in affected individuals [33], but few data are available about BKVAN; it probably due to recent emerging of this disease as an important cause of allograft failure following renal transplantation. BKV urinary shedding of infected urothelial cells occurs in 10 to 60% of renal transplant recipients [34] and literature data suggest that prospective monitoring of patients at risk for BKVAN may identify those with active infection before renal function deteriorates [35-37]. Recent studies demonstrated that BKVAN develop in as many as 8% of renal allograft recipients, with as many as 50% of patients experiencing graft loss over the next 2 to 3 years of follow-up [34,38,39]. A current study performed by Giraldi et colleagues show that, in a cohort of the 117 patients followed up every three months during a two year period after transplantation, 4 had BKVAN (3.4%) confirmed by quantitative assays on plasma and urine and assessed by allograft biopsy [40].

## BKVAN diagnosis

BKVAN diagnosis is very difficult since this disease is often misdiagnosed as acute rejection or drug toxicity. Diagnostic tools available include histopathology by means of renal allograft biopsy, detection of BKV DNA on plasma and urine by polymerase-chain-reaction (PCR) and quantitative PCR (QPCR) and presence of "decoy cells" in the urine sediment. Diagnostic confirmation may be obtained using IHC, in situ hybridization (ISH), and/or electron microscopy (EM) in renal biopsy specimens [34,41-45].

Early identification provides the opportunity for intervention with reduction of the immunosuppression in an effort to control BKV replication and prevent BKVAN. The risk factors predisposing to BKVAN appear to be multiple, with immunosuppressive regimens containing tacrolimus and MMF representing recognized associations [41,46]. Several investigators have begun to define risk factors for BKV disease among renal transplant recipients. The serologic status of the donor and the recipient appears to be a predictor of BKV infection, but it is not currently clear whether it influences the development of BKV nephritis. Tubular injury could be a factor promoting viral replication in an immunocompromised state induced by tacrolimus or MMF. The load of dormant BKV in the grafted organ is likely to be another important risk factor: no dormant virus, no re-activation and most likely, no BKVAN [47]. On these basis, since no specific anti-viral therapy is available, reduction in immunosuppression remains the mainstay of treatment with an increased risk of subsequent rejection. Therefore an accurate diagnosis is important, as it allows for early intervention and possible recovery of renal function.

Urine cytology is based on decoy cells recovery. Decoy cells are epithelial cells with enlarged nuclei and large basophilic ground-glass intranuclear viral inclusions, screening for their presence provides a simple and an inexpensive tool for the diagnosis of BKV nephropathy, nevertheless, Papanicolaou-stained urine sediment is not to be considered a specific morphological marker of BKV disease [48,49].

**Figure 2:**
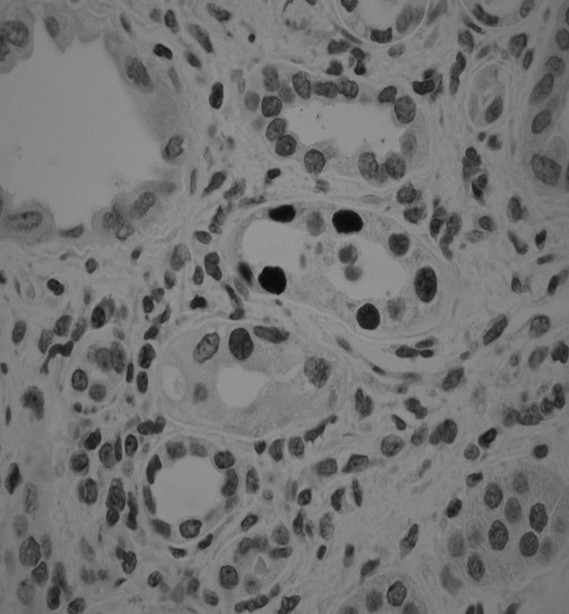
Immunohistochemistry, peroxidase stain, diaminobenzidine as marker, staining for BK polyoma virus with the antibody targeting the SV40 antigen. Note easily detectable, strong nuclear immunoreactivity in tubular cells. (350×), (41).

Electron microscopy is very sensitive for detection of BK virions, but the finding of viral particles is not diagnostic of BKVAN, since the ultrastructural appearance of BK virus is poorly typical. Virions are arranged in paracrystalline arrays of naked, round, electron-dense structures that measure 45 nm in diameter. It is important to emphasize that electron microscopy cannot distinguish BKV from JC virus [41] (Fig. 2).

The histological diagnosis of BKVAN requires evaluation of a renal biopsy with demonstration and confirmation of the polyomavirus cytopathic changes by IHC and ISH [41]. BKVAN is characterized by the presence of polyomavirus cytopathic changes in the epithelium of the renal tubules and urothelial lining. The infected cells have an enlarged nucleus with a gelatinous basophilic inclusion resulting from the accumulation of the newly formed virions [50]. Confirmation of the polyomavirus infection is usually performed with immunohistochemical stains for the simian virus 40 (SV40) large T antigen (AgT), which identifies all polyomavirus infections due to cross-reactivity between SV40 and both BKV and JCV. Distinction between the different types of polyomavirus requires the use of species-specific antibodies, ISH or in situ PCR. Systematic studies comparing the clinical utility of each method have not been performed [50]. The sections are stained with hematoxylin-eosin and examined by means of light microscopy in order to evaluate the integrity of the tissue before proceeding to molecular analysis, to identify possible pathologic changes, and in particular to search for the presence of morphologic equivalents of cellular polyomavirus infection. In situ hybridization and immunohistochemistry are carried out to define the viral status of the infected tissues The reactions are detected by means of the streptavidin-biotin method and are revealed using diaminobenzidine as a chromogen. In situ hybridization is performed to localize the nucleic acid sequences of BKV and JCV at the subcellular level using commercially available biotinylated DNA probes [51].

For efficient early diagnosis of BKVAN, various molecular approaches are recommended. Quantitative PCR is a non-invasive method clinically useful since it is high sensitive and specific and it supplies quantitative data that allow pharmacological therapy management by clinicians because specific antiviral therapy for BKVAN does not currently exist and the reduction in immunosuppression depend on viral loads in urine and plasma specimens of kidney transplants [32,33,36,52]. Nevertheless it is important to underlie that the relationship between BKV viruria and viremia, the cut-offs and predictive values of BKV viruria and viremia for the occurrence of BKVAN, are still largely undefined [33]. In fact some literature studies from 2004 to nowadays showed that measurements of BKV viruria and BKV viremia have a different prognostic value for patient's therapeutic response and duration of therapy. In accordance with Drachenberg et colleagues BKV viruria precedes BKV viremia and it is a prerequisite for histologically proven BKVAN because the viral replication within the graft finally leads from viruria to viremia [53]. This hypothesis is also sustained by other Authors that maintained that viremia is not present in patients with low-level/limited viral replication in the urinary tract [34,43,44,52,54]. Moreover, in relation to these Authors, viremia is not useful for screening because of blood inhibitors present in plasma sample. Finally, although analytical and physiological variations may be significant when comparing viral urine load in patients with BKVAN, there is general agreement that repeated values above 10^7 ^BKV copies per milliliter are associated with BKVAN [32,53]. On the other hand a recent study performed by Basse et collaborators suggested that BKV viremia is a rare event after renal transplantation but it has emerged as the most specific test for BKV associated nephropathy [55]. Some Authors retain BKV viremia as the standard for BKVAN diagnosis since the presence of the virus in the blood represents a significant tissue damage and confirm the renal parenchymal involvement [37,56]. Therefore serial determinations of BK viremia are the best tool to demonstrate resolution of the disease after immunosuppression has been decreased [37,55-58]. Nevertheless, a study carried out by Hymes et colleagues from June 2003 to January 2006 on 20 renal transplant children showed that most patients remained PCR-positive despite reduction of immunosuppression. Moreover they did not identify any one drug as more prevalent among patients with BK viremia [59].

## Conclusion

In conclusion, there are several aspects of BKVAN pathology in kidney transplant patients requiring evaluation; it includes BKV transmissibility within kidneys transplanted, target organ effects, risk factors, time frame of reactivation and the best treatment options. Therefore it is essential to understand and to monitor the delicate balance between viral infection, immune regulation in the transplant population and immunosuppressive therapy in order to minimize viral injury and rejection risk to patients with BKV infection. Measuring of BKV DNA in urine and serum is an useful and non invasive tool for early detection and monitoring, nevertheless a combined approach of molecular techniques must be utilized to identify BK virus-associated nephropathy at an early phase facilitating well timed clinical intervention.
